# A preliminary evaluation of a group compassion-focused intervention for individuals affected by perinatal loss

**DOI:** 10.3389/fpsyt.2025.1642423

**Published:** 2025-11-28

**Authors:** Rebecca Hunter, Allison Pilling, Hannah Warren, Katie Fox, Moninne McCormack

**Affiliations:** 1 Cheshire and Merseyside Maternal Mental Health Service, Mersey Care NHS Foundation Trust, Thomas House, Hollins Park Hospital, Winwick, United Kingdom; 2 University of Liverpool, Department of Primary Care and Mental Health, Liverpool, United Kingdom

**Keywords:** maternal mental health, perinatal grief, compassion, bereavement, group therapy, online, virtual

## Abstract

The United Kingdom (UK) National Health Service’s (NHS) long term plan established Maternal Mental Health Services (MMHS) to provide specialist psychological assessment and intervention for perinatal loss, birth trauma, and fear of childbirth. Perinatal loss increases the risk of psychological and grief-related distress. Compassion-Focused Therapy (CFT) is designed to reduce self-criticism and enhance emotional regulation and has shown promise in supporting perinatal populations. This study aimed to evaluate an online group CFT intervention delivered in an MMHS for individuals after perinatal loss. The study includes data from seven groups. In total, 30 women attended a 10-week CFT for perinatal loss psychological intervention group. The group was facilitated online and included psychoeducation and CFT skills to support with perinatal grief. Participants completed a range of self-report measures pre- and post-intervention that assessed self-criticism and self-compassion, symptoms of perinatal grief, psychological distress, and posttraumatic stress disorder symptoms. Participants demonstrated statistically significant improvements across all outcome measures following the intervention. Psychological distress decreased (*B* = −7.84, *p* <.001, *d* = 1.06), as did post-traumatic stress symptoms (*B* = −17.80, *p* <.001, *d* = 1.21), grief-related distress (*B* = −16.92, *p* <.001, *d* = 1.00), and self-criticism (*B* = −7.24, *p* = .006, *d* = 0.73). Goal-based outcomes improved significantly (*B* = 5.48, *p* <.001, *d* = 2.58). Effect sizes indicated large and clinically meaningful change across key domains. This study provides valuable insights into the role of CFT in supporting bereaved mothers within MMHS settings. The findings support preliminary evidence of the utility of CFT for perinatal loss. Future research can build on this by replicating with larger samples to further explore efficacy and incorporate assessment of long-term change. Acceptability within diverse samples also requires exploration.

## 1 Introduction

Perinatal mental health difficulties are estimated to affect 25.8% of women in England (1). These difficulties can include conditions such as depression, anxiety, and Post-Traumatic Stress Disorder (PTSD), which may arise during pregnancy or in the year following birth. Experiences of perinatal loss can disrupt women’s sense of identity as they navigate changes in how they view themselves as mothers, partners, and individuals (2). Spousal and family relationships may also be strained, with some women reporting increased conflict, emotional distancing, or difficulties in communication following a perinatal loss (3). Common symptoms of perinatal mental health difficulties include intrusive thoughts, hypervigilance, changes in mood, and heightened anxiety, all of which can significantly impair daily functioning (1).

The National Health Service (NHS) long term plan (4) committed to increasing access and availability to Specialist Perinatal Mental Health Services (SPMHS) for women across the United Kingdom (UK), including the creation of Maternal Mental Health Services (MMHS). There are currently 41 MMHS services operating across the UK (5) delivering life-changing care to women and their families following perinatal loss. This service evaluation was conducted to evaluate a specific intervention delivered within one MMHS. Understanding how these interventions perform in routine care is essential to inform ongoing development, adaptation, and scaling of support for individuals affected by perinatal loss.

Perinatal loss refers to the death of a baby during pregnancy, birth, or shortly after delivery, and encompasses a range of experiences including miscarriage, stillbirth, neonatal death, and other pregnancy losses such as ectopic, molar, or chemical pregnancies (6, 7). Miscarriage typically refers to loss before 24 weeks’ gestation, while stillbirth is defined as fetal death occurring at or after 24 weeks. Neonatal death refers to the death of a baby within the first 28 days of life. Globally, perinatal loss is a common experience, with an estimated 14–20% of pregnancies ending in miscarriage and approximately 2.6 million stillbirths occurring each year (8, 9). In the UK, miscarriage is the most frequently reported type of perinatal loss, affecting around 1 in 4 pregnancies (10–12). Stillbirth rates in the UK are approximately 3.9 per 1,000 births, and neonatal death occurs in 3.0 per 1,000 live births (13).

The prevalence of perinatal loss is not evenly distributed across populations. UK-based data indicate significant disparities in outcomes by ethnicity: stillbirth rates are twice as high for babies of Black ethnicity and 60% higher for babies of Asian ethnicity compared to those of White ethnicity (14, 15). These inequalities reflect broader social determinants of health and underscore the need for targeted support and intervention. For many women, perinatal loss, regardless of gestational age, can have profound and enduring psychosocial impacts (10, 16).

Perinatal loss can be devastating and traumatic, with profound psychological consequences for women and their families (17). Experiencing loss during the perinatal period is a well-established risk factor for the development of mental health difficulties, significantly increasing the likelihood of depressive symptoms, anxiety, PTSD and complicated grief in the parents (10). Compared with other forms of bereavement, perinatal loss is more likely to result in complicated grief (16). Complicated grief involves intense and persistent grief that extends beyond culturally accepted timeframes and causes significant functional impairment (18). The impact of perinatal loss can be far reaching, influencing decision making around about future pregnancies and negatively affecting spousal relationships (3). Furthermore, societal misconceptions and stigma, poor social support, and poor clinical care may exacerbate negative psychological impacts after perinatal loss (16).

Perinatal loss may not always be acknowledged within society, which can leave individuals feeling isolated and alone (19). In one study, 72.9% of women reported that their grief was perceived as invisible by society (20). This lack of societal recognition can give rise to disenfranchised grief (21), a form of grief influenced by social norms and expectations. Disenfranchised grief arises when there is a disconnect between an individual’s internal emotional experience and culturally accepted expressions of mourning (21). In the context of perinatal loss, this often means that grief is neither publicly acknowledged nor socially validated, leaving individuals unable to openly mourn their loss (22).

The needs of women following a perinatal loss are highly individual and diverse. Nonetheless, avoiding discussing the loss is often perceived negatively by women, as many women wish for their babies to be acknowledged and treated with dignity (23). Cultural beliefs and practices significantly shape how families grieve after the loss of a baby, influencing mourning rituals, the duration of grief, and the ways in which it is expressed. Although grief is a universal human experience, its expression and course can vary widely across cultural contexts (23). Supportive relationships play a critical role in promoting positive psychological adaptation after such profound loss (24, 25). Peer support and connection with others who have experienced similar losses have been identified as key to facilitating emotional expression and fostering a sense of empowerment (17, 23).

At present, there is a lack of guidance on the most effective psychological interventions to support women and families following perinatal loss. The National Institute for Health and Care Excellence (NICE) recommends that individual experiencing PTSD after a loss, should be offered high intensity psychological therapy for PTSD, specifically Cognitive Behavioural Therapy (CBT) or Eye Movement Desensitisation and Reprocessing (EMDR; 26). However, this guidance is not specific to perinatal loss and does not consider cultural or social meanings associated with this type of bereavement. Emerging practice-based evidence suggests that Compassion Focused Therapy (CFT) may be a promising therapeutic approach to support women experiencing psychological distress in the perinatal period (27).

CFT is an integrative, transdiagnostic psychological model developed to support individuals experiencing high levels of shame and self-criticism (28). Compassion is defined as, “*the sensitivity to suffering in self and others (engagement), with a commitment to try to alleviate and prevent it (action)”* (29, p. 19). Cultivating self-compassion is thought to help individuals navigate adverse life events, protect against self-critical thinking, and enhance overall psychological well-being (30, 31). A growing body of evidence indicates that CFT may be helpful for treating a wide range of mental health conditions (32). A meta-analysis showed that CFT is associated with increased self-compassion and mindfulness, as well as reductions in anxiety, depression, and psychological distress (33). Moreover, Lennard (34) demonstrated that a brief online self-compassion intervention improved depressive and PTSD symptoms in postnatal mothers, highlighting the potential utility of CFT in the perinatal period.

Although CFT is not currently included in NICE guidance for the clinical management of antenatal and postnatal mental health (35), a growing body of research suggests that CFT adapted for perinatal populations may be an effective approach for perinatal mental health difficulties (33, 36). For example, group-based CFT has been shown to reduce self-criticism, improve self-compassion, strengthen parent-infant bonding, and support individuals in achieving personalized goals in the perinatal period (27). Additionally, brief and online CFT interventions have demonstrated preliminary effectiveness and accessibility (34, 37), providing viable alternatives for individuals facing barriers to in-person therapy, such as childcare responsibilities or physical recovery post-birth. These findings highlight the potential utility of CFT for maternal populations; however, the impact of more comprehensive CFT interventions in this context remain underexplored.

There is growing interest in the broader application of CFT within maternal mental health, particularly due to its potential to benefit both parents and their children (38–41). A notable gap in the literature concerns the use of CFT for individuals who have experienced perinatal loss, a distinct and significant population within the newly commissioned MMHS in the UK. Despite the well-documented psychological impact of perinatal loss, there remains a lack of targeted, evidence-based interventions to support this group effectively.

To address this gap, a 10-session, online, group-based CFT intervention was developed and piloted within the Cheshire and Merseyside MMHS. The current study aims to evaluate changes in well-being following participation in this group intervention for women who have experienced perinatal loss.

## 2 Context (setting and population)

The context for the current study is the Cheshire and Merseyside NHS MMHS which is commissioned to provide psychological treatment and support to women experiencing moderate to severe psychological distress in the context of perinatal loss. Use of Routine Outcome Measures (ROMS), alongside psychological assessment, support clinical decision making around moderate to severe clinical presentation. The service provides psychological therapy and specialist midwifery interventions, alongside peer support.

The CFT for perinatal loss group intervention was developed by a Clinical Psychologist with consultation from people with lived experience and local voluntary and third sector organisations who had experience of running groups for perinatal loss. The group was part of the clinical pathway for individuals who were either parenting or pregnant after a loss and experiencing difficulties in their grief and adjustment, including feelings of shame, self-criticism, and self-blame. To be eligible for the group intervention, participants were required to have experienced at least one perinatal loss (defined as miscarriage, stillbirth, or neonatal death) and to be experiencing moderate to severe psychological distress, including difficulties such as persistent grief, anxiety, low mood, or trauma-related symptoms. Exclusion criteria included any presentation outside the service remit (e.g. active psychosis, current severe substance misuse, or acute risk of harm requiring crisis intervention). Participants were recruited into the group following an assessment with a clinician from the MMHS where their intervention options were discussed and a care plan agreed.

The average time since participants’ most recent perinatal loss was 26.2 months, with a range of 2–116 months. Although many participants were outside the 12-month perinatal period, they continued to experience psychological distress related to their loss that was significantly impacting their functioning. This heterogeneity reflects the service’s referral pathway and the enduring psychological needs of this population.

Participants completed an individual pre-group assessment, which included completion of ROMS (serving as baseline data), discussion of treatment goals, and the opportunity to ask questions about the group. Following the 10-session group, an individual post-group review was conducted to repeat ROMS, evaluate progress, and determine whether further intervention was required. The procedure was therefore: pre-treatment individual session, 10-session group protocol, post-treatment individual session.

The intervention was delivered virtually via Microsoft Teams and facilitated by three health-care professionals, including at least two psychological practitioners (e.g. Clinical Psychologist or Psychological Therapist) and either an Assistant Psychologist or a Peer Support Worker. All facilitators received training on the intervention prior to delivery. To support treatment fidelity and ensure consistent delivery, weekly supervision was provided by a clinical psychologist throughout the intervention period. Supervision offered space to reflect on group dynamics, reinforce adherence to the therapeutic model, and address any challenges in implementation. While formal fidelity checklists were not used, this structured support aimed to maintain reliability in the delivery of the intervention within the context of NHS service evaluation.

Sessions were held weekly for 10 weeks, with each session lasting 2 hours. Content was delivered using PowerPoint slides, videos, handouts, and mindfulness exercises, and participants were encouraged to practice skills between sessions to reinforce and apply their skills. Individuals who missed a session were offered a one-to-one catch-up to cover the missed content.

The intervention was informed by CFT and tailored to support individuals experiencing psychological distress following perinatal loss. Each session followed a structured format comprising psychoeducation, experiential practice, reflective discussion, and take-home exercises. Sessions began with grounding techniques, such as soothing rhythm breathing, and closed with compassion-focused imagery or mindfulness practices to consolidate learning and promote emotional regulation.

CFT principles were introduced progressively, beginning with the three emotion regulation systems (threat, drive, and soothe), understanding self-criticism, and cultivating the compassionate self. These concepts were contextualised within the lived experience of perinatal loss, with attention to disenfranchised grief, identity disruption, and the loss of the assumptive world. For example, Session 2 explored the emotional impact of secondary losses, while Session 4 addressed social norms around grief and introduced Tonkin’s model of grief (42) to validate non-linear grieving processes.

Experiential techniques included compassionate imagery (e.g., compassionate place, compassionate self), chair work, compassionate letter writing, and re-scripting of trauma-related nightmares. These were adapted to support grief-specific needs, such as maintaining continuing bonds, processing flashbacks, and responding to self-blame with compassionate self-correction. Participants were invited to engage with symbolic representations of their loss, and to explore multiple emotional states and selves with empathy and validation. Sessions also incorporated practical tools such as the developing a compassionate kit bag, thought diaries, and gratitude practices, designed to foster resilience and self-soothing. The final sessions focused on integrating compassionate practices into daily life, strengthening group support, and planning next steps. **Table 1** provides a detailed overview of session themes and take-home messages.

**Table 1:** CFT group content.

Session	Session content	Take home messages
Session 1	Introductions, group rules, creating safeness, hopes and fears, goals. Understanding emotions, grief, unhelpful coping mechanisms, perinatal loss, empathy vs sympathy. Soothing rhythm breathing practice.	What is grief? What does grief look like?
Session 2	Me and my baby, psychoeducation of the brain and mindfulness, the definition of compassion and what compassion aims to do, loss of the assumptive world, secondary losses, peer support. Mindfulness to breath, sounds, and body practice.	What is compassion? Why compassion? The loss of the assumptive world.
Session 3	Brain and compassionate loops, old and new brain, mindful compassion, three flows to compassion, blocks to self-compassion, compassionate first aid kit. Mindfulness to thoughts practice.	Developing a compassionate mind. three flows of compassion. Barriers to compassion.
Session 4	Social norms around grief, three circle model of emotion regulation, continuing bonds, Tonkins model of grief, compassionate kit, compassionate smell. Compassionate place practice.	Social norms and culture. Three systems of grief. Tonkin’s Model of Grief.
Session 5	Understanding our story with compassion, trauma memory, brain model of PTSD, flashbacks and nightmares, Nightmare imagery re-scripting. Compassionate self practice.	Trauma and the brain. Flashbacks and Nightmares.
Session 6	Compassionate voices, who am I, multiple emotions, multiple selves, communication and empathy, the compassionate self and threat emotions, developing a compassionate being. Compassionate image practice.	Compassionate self and the threat emotions. Compassionate imagery.
Session 7	Self-criticism, threats and fears, blame, experiencing the critic, moving from a critical to a compassionate self-corrector. Relating to different parts of ourselves from a compassionate mind practice.	Self-blame. Inner critic. Moving from a critical to a compassionate self-corrector.
Session 8	Chair practices, compassionate reframe, compassionate thought diary, gratitude diaries, taking in the good, stages of acceptance. Soften, soother, allow practice.	Critic. Responding in a different way.
Session 9	Gratitude diaries, thought diaries, making sense of frightening thoughts, compassionate letter writing, the power of vulnerability. Compassion under the duvet practice.	Compassionate letter writing.
Session 10	Compassionate letters, day in life of self-compassionate at best, the power of vulnerability, continuing bonds, support, next steps. Compassion to the group practice.	Support and next steps.

ROMS were used to evaluate the group and were administered pre-group (within 4 weeks prior to the group starting) and post-group (within 4 weeks of the group finishing) to evaluate changes in participant’s symptomology.

### 2.1 Forms of self-criticizing/attacking and self-reassuring scale

The FSCRS is a clinically validated 24-item self-report questionnaire which measures how critical/attacking and how supportive/reassuring people are when things go wrong for them. The FSCRS has three subscales 1) inadequate self, 2) reassured self, and 3) hated self. The scale is measured from 0 (not at all like me) to 4 (extremely like me). Items include “I have a sense of disgust with myself” and “I think I deserve my self-criticism”. Scores range from 0 to 88. Higher scores indicate higher self-criticism and/or self-reassuring. The FSCRS has demonstrated strong internal consistency, with Cronbach’s alpha values ranging from .86 to .90 across subscales (43). Test-retest reliability has also been reported as acceptable, with a correlation of .88 over a two-week period (43). Construct validity is supported through significant correlations with depression, anxiety, and self-compassion, consistent with theoretical expectations (43). Self-compassion was assessed using the Reassured Self subscale of the FSCRS, which captures the ability to respond to personal setbacks with warmth, reassurance, and emotional support. This measure was introduced as part of the group halfway through this service evaluation period, which explains why only half the sample have completed this measure.

### 2.2 Perinatal grief scale

The PGS is a clinically validated 33-item questionnaire measuring symptoms of grief in relation to loss around pregnancy. The PGS has three subscales 1) active grief, 2) difficulty coping, and 3) despair. The scale is measured from 1 (strongly agree) to 5 (strongly disagree). Items include “I am grieving for the baby” and “I feel empty inside”. Scores range from 11 to 55. Higher scores indicate higher symptoms relating to grief. The PGS has demonstrated excellent internal consistency, with Cronbach’s alpha reported as.97 for the full scale and subscale values ranging from.87 to.95 (44). Test-retest reliability has been found to be strong, with a correlation of.83 over a one-month interval (44). Construct validity is supported through factor analysis and significant correlations with measures of depression, anxiety, PTSD, and self-compassion (44).

### 2.3 Clinical outcomes in routine evaluation-10

The CORE-10 is a clinically validated 10-item questionnaire measuring symptoms of distress in the form of anxiety, depression, trauma, physical problems, functioning and risk to self in the last seven days. The CORE-10 has six problem domain items, three functioning domain items and one risk item. The scale is measured from 0 (not at all) to 4 (most or all of the time). Items include “I have felt tense, anxious or nervous” and “I have felt panic or terror”. Scores range from 0-40. Higher scores indicate higher psychological distress. The CORE-10 has demonstrated good internal consistency, with Cronbach’s alpha values ranging from.80 to.90 (45). Test-retest reliability has been reported as strong, with an intraclass correlation coefficient of.83 over a two-week period (45). Construct validity is supported by confirmatory factor analysis and moderate to strong correlations with other validated measures of psychological distress (45).

### 2.4 PTSD checklist 5

The PCL-5 is a clinically validated 20-item questionnaire, measuring symptoms of PTSD in the last month. The scale is measured 0 (not at all) to 4 (extremely) on how the person has felt in the last month in relation to a traumatic experience. Scores of >31 indicate the presence of PTSD symptoms. Scores range from 0 to 80. Higher scores indicate higher PTSD symptomology. Items include “How much have you been bothered by repeated, disturbing, and unwanted memories of the stressful experience?” and “How much have you been bothered by feeling very upset when something reminded you of the stressful experience?”. In this context, the stressful experience was the perinatal loss. The PCL-5 has demonstrated excellent internal consistency, with Cronbach’s alpha typically reported around.94 (46). Test-retest reliability is strong, with temporal stability correlations exceeding.80 (46). Construct validity is supported through robust correlations with depression, anxiety, and functional impairment, and confirmatory factor analyses have validated its latent structure (46).

### 2.5 Goal based outcomes

Goal Based Outcomes (GBOs) are a way to evaluate progress towards goals in clinical work with individuals. Goals are rated from 0 (no progress towards the goal) and 10 (goal has been reached fully). Participants were asked at baseline to identify a goal(s) for the CFT group, which was then re-rated at the end of treatment. Test-retest reliability of GBO has been found to be acceptable over periods ranging from 6 to 24 weeks (47).

### 2.6 Analysis

Prior to hypothesis testing, baseline comparability across the seven intervention groups was examined via one-way ANOVAs. No statistically significant differences were found between groups in demographic characteristics or pre-intervention scores on relevant outcome measures, indicating comparability at baseline.

To assess the suitability of parametric tests, normality was evaluated through visual inspection of histograms and Q-Q plots, alongside skewness and kurtosis statistics. Shapiro-Wilk tests were conducted for each measure, and all values were non-significant, supporting the assumption of normality.

All analyses were conducted using SPSS version 27. To account for the hierarchical structure of the data, with participants nested within seven intervention groups, multilevel modelling was employed. This approach allows for random intercepts by group and appropriately adjusts for the non-independence of observations within clusters. To address the risk of inflated Type I error rates due to multiple comparisons, a Bonferroni correction was applied, adjusting the alpha level to.00555 (.05/9).

## 3 Results

A total of 30 women attended the CFT for loss group intervention between September 2022 and January 2025. A total of 7 groups were delivered with an average of 4.3 participants in each group (*SD=* 0.70, range 3-5). Participants attended an average of 8.4 sessions (*SD=* 1.81, range 5-10). Participant demographics can be seen in **Table 2**.

**Table 2:** Participant demographics.

Variable	Value
Age	*M = 33.4, SD = 5.32, range = 22–42*
Ethnicity	
White British	28 (93.3%)
Asian or Asian British	1 (3.3%)
Black or Black British	1 (3.3%)
Type of perinatal loss	
Miscarriage (≤14 weeks gestation)	8 (26.7%)
Late miscarriage (14–24 weeks gestation)	3 (10.0%)
Medical termination	1 (3.3%)
Stillbirth (≥24 weeks gestation)	13 (43.3%)
Neonatal or infant death	5 (16.7%)
Time since most recent loss (months)	*M* = 26.2, *SD* = 23.88, range = 2–116

*M*, mean; *SD*, standard deviation.

To examine changes in psychological outcomes from pre- to post-intervention, a series of mixed-effects linear models were estimated for each measure, with time (pre and post intervention) entered as a fixed effect and participants included as a random intercept to account for repeated observations. All models converged normally. **Table 3** presents the multilevel model results, including unstandardized coefficients, standard errors, 95% confidence intervals, and intraclass correlation coefficients (ICCs).

**Table 3:** Multilevel model results.

Measure	N	Pre-Group Mean (SD)	Post-group Mean (SD)	B (Post–Pre)	SE	95% CI	p	Bonferroni adjusted p	Mean difference	Cohen’s d	ICC
CORE-10	29	23.13 (6.29)	15.14 (8.35)	−7.84	1.32	[−10.43, −5.25]	<.001	<.001	−7.69	−1.06	.51
PCL-5	30	53.17 (11.07)	35.37 (19.05)	−17.80	2.64	[−22.98, −12.62]	<.001	<.001	−17.00	−1.21	.55
PGS Total	28	125.73 (14.98)	108.96 (25.00)	−16.92	3.11	[−23.01, −10.83]	<.001	<.001	−17.00	−1.00	.66
FCSRS Total	15	45.24 (8.67)	38.13 (8.03)	−7.24	2.62	[−12.37, −2.11]	.006	0.051	−7.87	−0.73	.18
GBO1	23	1.65 (1.21)	7.11 (2.14)	5.48	0.42	[4.65, 6.31]	<.001	<.001	5.57	2.58	.21

Estimates are from mixed-effects linear models with random intercepts for participants, coding time (0 = pre, 1 = post). *B* represents the estimated mean change from pre- to post-intervention. All p-values correspond to the fixed effect of time. *Cohen’s d* values are calculated from paired differences. ICC, intraclass correlation coefficient (proportion of total variance at the participant level).

Analyses indicated statistically significant improvement across all outcome measures. Participants showed a significant reduction in psychological distress (*B* = −7.84, SE = 1.32, 95% CI [−10.43, −5.25], *p* <.001), representing a large within-subject effect (*d* = 1.06). Similarly, symptoms of post-traumatic stress symptoms decreased significantly (*B* = −17.80, SE = 2.64, 95% CI [−22.98, −12.62], *p* <.001, *d* = 1.21). Grief-related distress also decreased significantly following the intervention (*B* = −16.92, SE = 3.11, 95% CI [−23.01, −10.83], *p* <.001, *d* = 1.00). Self-criticism also decreased significantly (*B* = −7.24, SE = 2.62, 95% CI [−12.37, −2.11], *p* = .006, *d* = 0.73). In contrast, GBO improved significantly (*B* = 5.48, SE = 0.42, 95% CI [4.65, 6.31], *p* <.001), with a large effect (*d* = 2.58). After applying a Bonferroni correction to control for multiple comparisons (adjusted α = .00555), all outcomes except the FSCRS Total remained statistically significant. The reduction in self-criticism on the FSCRS Total approached significance (p = .00568) but did not meet the adjusted criterion. The pattern of results, however, still indicates large and consistent improvements across all measures, with particularly robust effects on distress (CORE-10), trauma symptoms (PCL-5), grief (PGS Total), and goal attainment (GBO1).

ICCs ranged from.18 to.66, indicating that between-participant variability accounted for a moderate proportion of total variance across outcomes. Together, these findings suggest that participants experienced significant improvements in distress, trauma symptoms, grief, and self-criticism, alongside substantial progress toward personal goals following the intervention.

At the end of the group intervention, 26 participants (87%) were discharged from the MMHS and 4 (13%) went on to have further individual psychological therapy within the service.

## 4 Discussion

This study aimed to evaluate a group CFT intervention for women who had experienced perinatal loss in a MMHS. To the authors’ knowledge, it is the first study to examine CFT for this specific population, contributing to the growing evidence base for CFT interventions in perinatal populations and within NHS MMHS settings.

The findings provide preliminary support for the intervention in improving psychological well-being. Across all measures, participants demonstrated significant pre–post improvements, including reductions in distress, trauma symptoms, and self-criticism, as well as notable decreases in grief-related distress. The marked improvement in goal-based outcomes suggests that participants perceived meaningful personal progress in areas they identified as important at baseline. The results suggest that group CFT may be an effective approach for addressing the emotional needs associated with perinatal loss. As this is the first service evaluation to explore group-based CFT for perinatal loss, it is not possible to compare these findings to other studies. However, this is supported by findings from Lawrence (27), who found group-based CFT intervention to support women in the perinatal period more broadly. In addition, concerning PTSD symptoms, there were significant reductions following the group intervention. Up to 39% of women will develop PTSD symptoms following a perinatal loss (12) and previous research suggests CFT to be effective at reducing PTSD symptoms after birth (48). The current findings therefore show promise for further implementation of the group-based CFT intervention within MMHS to support women following perinatal loss.

The CFT group offers a psychological intervention that is easy to facilitate, with the potential to reduce waiting times and support timely access to intervention. In addition, retention in the group was generally good. Further research should seek to build on this by incorporating a qualitative component to better understand what was valued most by women within the group and their perceptions of acceptability.

Perinatal loss can pose a significant risk to maternal mental health and research suggests that perinatal loss impacts subsequent mental health outcomes (10, 49). Following a perinatal loss, parents may experience sadness, guilt, anxiety and depressive symptoms as part of their grief and mourning experience. The length of time that a parent may experience this will differ by individual and culture, but some research suggests this can last between 1–2 years (50). For some, this experience can be complicated by prolonged symptoms which may require psychological treatment (50). An important consideration in interpreting the findings is the variability in time since participants’ most recent perinatal loss. While the average was 26.2 months, some losses occurred as long as 116 months (9.5 years) prior. This means that many participants were outside the conventional 12-month perinatal period commonly used to define the perinatal timeframe. It is possible that women further from their perinatal loss may have had different treatment needs or responses compared with those more recently bereaved. For some, unresolved grief may have persisted over many years, whereas for others, later life stressors or subsequent pregnancies may have reactivated earlier experiences of loss.

This group and evaluation are written from a White Eurocentric perspective, and cultural beliefs and practices significantly shape how families grieve after the loss of a baby, influencing mourning periods, rituals, and the expression of grief. Not pathologizing normal grief and mourning reactions is an important consideration for MMHS and psychological assessment by appropriately trained professional is required to understand whether psychological treatment is indicated.

It should also be noted that the current study design cannot establish causality regarding whether the psychological distress observed was attributable solely to perinatal loss. Other factors, such as concurrent life events, pre-existing mental health difficulties, or relationship stressors, may also have contributed to participants’ presentations. Consequently, our findings should be interpreted as evidence of the acceptability and potential clinical value of a CFT group intervention in a heterogeneous sample, rather than as proof of efficacy in directly treating perinatal loss-related distress. Future research using controlled or longitudinal designs will be needed to examine causal pathways and to explore how time since loss influences treatment outcomes. Incorporating control or comparison groups would allow for stronger conclusions regarding treatment effects and help isolate active therapeutic mechanisms. Where feasible, randomisation and careful sequencing of outcome measurement could support temporal precedence and reduce bias. Additionally, future studies should consider extending the intervention to non-gestational or non-birthing partners, who may also experience profound grief following perinatal loss. Exploring the applicability and impact of CFT for these individuals would enhance the inclusivity and relevance of the intervention across family systems.

This study identified significant pre–post improvements across several outcome measures following the intervention. However, given the number of primary analyses conducted (n = 9), we applied a Bonferroni correction to mitigate the risk of inflated Type I error rates. Under this more stringent threshold (α = .00555), most effects remained statistically significant, however, reductions in self-criticism on the FSCRS Total did not meet the adjusted criterion. These findings should be interpreted in light of the exploratory nature of the analyses and the absence of an *a priori* power calculation. While the results are promising, they warrant replication in larger samples with pre-specified hypotheses and correction strategies. We have included effect sizes and confidence intervals to support interpretation beyond p-values alone.

## 5 Conceptual and methodological constraints

While the research provides valuable insights into the potential benefits of CFT in maternal mental health settings, there are several limitations that must be acknowledged. One limitation is the small sample size, making it difficult to generalize findings in terms of the wider population. In addition, the design limits the understanding of the long-term effects and any sustained impact of CFT on maternal mental health including its potential benefits for both parents and children (38, 40).

Another notable gap within the current research is the limited diversity within the sample. From the demographic data that were recorded, 93.3% identified as White British, 3.3% identified as Asian or Asian British and 3.33% identified as Black or Black British. Whilst the population is representative of those who typically access the service, it raises important concerns about the cultural relevance and applicability of CFT in more diverse populations as well as access to NHS services by women from different ethnic groups. An individual’s cultural beliefs and values may shape their perceptions of and responses to perinatal loss and it is essential that psychological interventions are culturally sensitive (19, 23, 51). The prevalence of perinatal loss is unequal across social groups and is significantly higher for black and other ethnic minority groups (14), however, only two women from ethnic minority groups accessed this group intervention over a 2-year period. While the intervention aimed to be inclusive and sensitive to diverse experiences of grief, we acknowledge that cultural expressions of grief vary widely and may influence how individuals engage with therapeutic content. Cultural norms shape not only emotional expression but also beliefs about loss, mourning rituals, and help-seeking behaviours. Future adaptations of this intervention should consider co-production with service users from minoritised communities to ensure cultural relevance, for example, by incorporating culturally meaningful metaphors, addressing systemic barriers to care, and offering flexible formats that accommodate different grieving practices. These disparities underscore the need for culturally responsive interventions that not only support individual healing but also acknowledge the social and systemic contexts in which grief occurs. More research is needed into the psychological and support needs of women from black, Asian and other ethnic minority groups following perinatal loss to help development of clinical pathways and services within MMHS.

This evaluation employed a single-group pre-post design without a control or waitlist comparison, which limits the ability to draw causal conclusions about the intervention’s effects. While observed improvements in psychological outcomes are encouraging, it is not possible to determine whether these changes were attributable to the intervention itself, its specific therapeutic mechanisms, or other factors such as spontaneous recovery, therapeutic engagement, or the supportive nature of group participation. This design reflects the pragmatic constraints of NHS service evaluation, where the priority is to assess feasibility and acceptability within routine care. Nonetheless, future research should incorporate controlled designs to more rigorously evaluate efficacy and isolate active components of the intervention.

To conclude, this study provides preliminary evidence that an online group CFT intervention can reduce psychological distress, PTSD symptoms, grief-related distress, and self-criticism while enhancing goal attainment in women following perinatal loss. The findings highlight the potential of CFT as a valuable therapeutic approach within MMHS settings. Future research should build on this with larger and more diverse samples, examine long-term outcomes, and explore acceptability.

## Data Availability

The authors will provide the raw data underlying this article’s conclusions upon request to the corresponding author.
